# Stage-specific rhizosphere microbial succession *is associated with* nutrient cycling in the desert plant *Leymus racemosus* (Lam.) tzvelev

**DOI:** 10.1186/s12870-026-09294-z

**Published:** 2026-06-20

**Authors:** Yufang Sun, Jinfeng Tang, Sijie Ma, Ailijiang Maimaiti, Jun Liu, Juan Qiu, Jie Ge

**Affiliations:** 1 Xinjiang Key Laboratory for Ecological Adaptation and Evolution of Extreme Environment Organisms, College of Life Sciences, Xinjiang Agricultural University, Urumqi, 830052 China; 2 College of Life Sciences, Xinjiang Agricultural University, Urumqi, 830052 China

**Keywords:** *Leymus racemosus*, Rhizosphere microbiome, Growth stage, Nutrient cycling, Microbial succession

## Abstract

**Background and aims:**

Plants regulate nutrient uptake and growth by recruiting rhizosphere microorganisms via root exudates. However, a systematic understanding of how the rhizosphere core and functional microbiota jointly regulate the dynamics of carbon, nitrogen, phosphorus, and potassium across the entire plant life cycle in desert ecosystems remains limited. In this study, we asked: how does the succession of rhizosphere bacterial communities align with stage-specific nutrient demands in the desert plant *Leymus racemosus*?

**Methods:**

We used 16 S rRNA high-throughput sequencing to analyze the rhizosphere bacterial communities and nutrient contents of the desert plant *Leymus racemosus* at three growth stages (seedling, flowering, maturity) in the Kalamaili Nature Reserve, Xinjiang, China. For each stage, ten 5 × 5 m quadrats (20 m apart) were established; 6–10 healthy plants were sampled per quadrat, and rhizosphere soil from each quadrat was pooled into one composite sample (*n* = 10 per stage).

**Results:**

*Arthrobacter*, identified as a core taxon, was associated with the stability of hydrolyzable nitrogen across all growth stages. *Bacillus* became the dominant genus during the flowering stage, based on correlation and functional prediction, it may contribute to nutrient supply, reflecting a potential “investment” strategy. At maturity, enhanced microbial cooperation (inferred from co-occurrence and correlation analyses) combined with reduced plant demand was associated with the accumulation of rhizosphere nutrients, possibly facilitating energy storage for subsequent growth. These findings provide a potential answer to our question, suggesting that the plant recruits distinct microbial alliances at different phenological phases—a persistent *Arthrobacter*-based system for nitrogen buffering, a transient *Bacillus*-enriched community for rapid nutrient mobilization at flowering, and a synergistic network at maturity for delayed nutrient accumulation.

**Conclusions:**

This study reveals the developmental dynamics of rhizosphere bacterial community assembly and nutrient regulation in *L. racemosus* and provides a theoretical basis for further elucidating plant–microbe interactions in desert ecosystems. However, the proposed functional roles of specific taxa are primarily derived from correlation and predictive analyses; experimental validation (e.g., strain isolation, inoculation tests, and metabolomics) is needed to establish causality.

**Supplementary Information:**

The online version contains supplementary material available at https://doi.org/10.1186/s12870-026-09294-z.

## Introduction

The plant rhizosphere microbiome comprises specific microbial communities, including primarily bacteria and fungi, that colonize the root surface and the surrounding soil zone [1]. Often referred to as the plant’s “second genome”, these microbes collectively form a complex rhizosphere system [2]. Through close interactions with plants, rhizosphere microorganisms directly or indirectly regulate plant growth and development [2, 3]. Rhizosphere microbes act as biotransformers of soil nutrients. Through their metabolic activities, they drive the transformation and cycling of key elements such as carbon, nitrogen, phosphorus, and potassium. In addition, they play indispensable roles in organic matter decomposition, as well as in nutrient solubilization and immobilization [4, 5]. Consequently, these microorganisms strongly influence soil nutrient bioavailability, thereby affecting plant nutrient uptake, growth, and development [6].

Numerous studies have shown that plant developmental stage is a key driver of rhizosphere microbial community succession [7, 8]. Plants move from the vegetative seedling stage to the reproductive flowering stage. During this transition, they actively recruit specific microbial taxa via root exudation to acquire nutrients needed at each developmental phase [9, 10]. For example, during periods of vigorous vegetative growth, microbial taxa associated with nitrogen fixation and transformation may be preferentially enriched. In contrast, functional microbial groups with high efficiency in phosphorus and potassium solubilization may be favored during the reproductive stage [7, 11–15]. This “on-demand” recruitment model highlights plants’ strong capacity for precise regulation of the rhizosphere microenvironment.

Although previous studies have provided insight into microbial functional traits associated with individual growth stages, a systematic understanding of how the core and functional microbiota interact across the entire plant life cycle remains lacking. Specifically, it is still unknown how their synergy co-regulates dynamic changes in nutrients such as nitrogen, phosphorus, and potassium [11]. To address this gap, we used *Leymus racemosus* as a model to investigate the following specific questions: (i) How do the rhizosphere bacterial community structure and soil nutrient contents change synchronously across the seedling, flowering, and maturity stages? (ii) Which bacterial genera act as core (persistent) vs. functional (stage-enriched) taxa, and what are their potential roles in nutrient cycling? (iii) Do the co-occurrence patterns among bacterial genera correlate with the observed nutrient dynamics, suggesting potential synergistic effects?

Based on previous observations, we formulated two exploratory hypotheses: (i) the relative abundances of core and functional taxa would shift in a pattern that mirrors stage-specific soil nutrient pools; (ii) co-occurrence networks among bacterial genera would become more complex at maturity, potentially driving delayed nutrient accumulation.

*Leymus racemosus* (Lam.) Tzvelev is a perennial grass species typical of the Junggar Desert in Xinjiang, China [16, 17]. It exhibits distinct phenological stages, including the seedling stage in May, the flowering stage in June, and the maturity stage in July. The above questions and hypotheses are investigated using this species, and our analyses aim to provide a systematic description of microbial–nutrient associations and to generate testable hypotheses for future mechanistic studies.

## Materials and methods

### Sample collection

Samples were collected from May to early July 2024 in a typical desert area of the Kalamaili Mountain Ungulate Wildlife Nature Reserve, located in the Junggar Basin of northern Xinjiang (89.082322°E, 45.246486°N), one of the main distribution areas of *Leymus racemosus*. This species is listed as a Class II protected plant in the Xinjiang Uygur Autonomous Region, and all necessary permissions for the collection of plant material in this field study were obtained from the Administration of the Kalamaili Mountain Ungulate Wildlife Nature Reserve, with the approval of the local government. The research complied with all relevant institutional, national, and international guidelines and regulations concerning plant collection and access to protected areas. The plant species was formally identified by Professor Juan Qiu based on morphological characteristics. A voucher specimen (Accession Number: 202407-SYS21) has been deposited in the Herbarium of Xinjiang Agricultural University.

*L. racemosus* grows in patchy populations across sandy habitats and is considered a dominant species in this region. A uniform patch with consistent growth vigor and similar plant cluster size was selected as the standardized sampling site. To accurately characterize the rhizosphere microbial community at each developmental stage and avoid interference among successive sampling events, independent and randomly distributed quadrats were established for each stage: seedling (**S**), flowering (**F**), and maturity (**M**). During each sampling event, ten 5 m × 5 m quadrats were arranged at 20 m intervals, and 6–10 healthy individuals were selected from each quadrat for sampling. For each growth stage (seedling, flowering, maturity), ten independent quadrats were sampled, resulting in ten biological replicates per stage (one composite sample per quadrat).

During sampling, root systems were carefully excavated, and loosely attached non-rhizosphere soil was gently shaken off, while tightly adhering rhizosphere soil was retained. The intact rhizosphere samples were placed in sterile sealed bags, immediately stored in ice-filled containers, and transported to the laboratory. Under aseptic conditions, rhizosphere soil was gently separated from the roots to obtain rhizosphere soil samples [18, 19]. For each quadrat, rhizosphere soil from all sampled plants was pooled into one composite sample. Each composite sample was divided into two portions: one was flash-frozen in liquid nitrogen and stored at − 80 °C for microbial diversity analysis, and the other was used to determine soil physicochemical properties.

### High-throughput sequencing

Total microbial genomic DNA was extracted from the rhizosphere soil samples using an E.Z.N.A.^®^ Soil DNA Kit (Omega Bio-Tek, Norcross, GA, U.S.), according to the manufacturer’s instructions. The quality and concentration of the extracted DNA were assessed by 1.0% agarose gel electrophoresis and a NanoDrop2000 spectrophotometer (ThermoFisher Scientific, United States) (A260/A280 ratio between 1.8 and 2.0; A260/A230 ratio > 1.8), respectively. Only samples meeting these criteria were used for subsequent library preparation, qualified DNA samples were stored at -80 °C for subsequent analysis.

The hypervariable V3-V4 region of the bacterial 16 S rRNA gene was amplified using the primer pair 338 F (5’-ACTCCTACGGGAGGCAGCAG-3’) and 806R (5’-GGACTACHVGGGTWTCTAAT-3’) on a T100 Thermal Cycler PCR system (Bio-Rad, USA) [20]. The resulting amplicons were purified, pooled in equimolar concentrations, and subjected to paired-end sequencing on an Illumina NextSeq2000 platform (Illumina, San Diego, USA) [21]. All sequencing procedures were performed by Majorbio Bio-Pharm Technology Co., Ltd. (Shanghai, China) following standard operational protocols. The raw sequencing data have been deposited in the NCBI Sequence Read Archive (SRA) under BioProject accession number PRJNA1241221. The SRA accession numbers are SRR35918480–SRR35918497 and SRR38507130–SRR38507141. The BioProject overview page is available at: https://www.ncbi.nlm.nih.gov/bioproject/PRJNA1241221.

### Analysis of soil physicochemical properties

Soil organic carbon (SOC) was determined using the potassium dichromate oxidation method. Total nitrogen (TN) was measured by acid digestion followed by the Kjeldahl method (ATN-300). Available nitrogen (AN) was quantified using the alkali-hydrolyzed diffusion method. Available phosphorus (AP) was extracted with sodium bicarbonate and measured using the molybdenum-antimony anti-spectrophotometric method. Total potassium (TK) was analyzed by alkali fusion followed by flame photometry (FP6431). Available potassium (AK) was extracted with ammonium acetate and measured using flame photometry (FP6431) [22, 23].

### Statistical analysis

Raw FASTQ files were initially demultiplexed using a custom Perl script. Sequence reads were then processed with fastp (v0.19.6) [24] for quality control and filtering, and assembled using FLASH (v1.2.7) [25] with the following criteria: (i) the reads were truncated at any site receiving an average quality score of < 20 over a 50 bp sliding window, and the truncated reads shorter than 50 bp were discarded, reads containing ambiguous characters were also discarded; (ii) only overlapping sequences longer than 10 bp were assembled according to their overlapped sequence. The maximum mismatch ratio of overlap region is 0.2. Reads that could not be assembled were discarded; (iii) Samples were distinguished according to the barcode and primers, and the sequence direction was adjusted, exact barcode matching, 2 nucleotide mismatch in primer matching. High-quality sequences were clustered into operational taxonomic units (OTUs) using UPARSE 7.1 with 97% sequence similarity level. The most abundant sequence for each OTU was selected as a representative sequence. singletons and chimeras were removed. The OTU table was manually filtered, i.e., chloroplast sequences in all samples were removed (v7.1) [26]. To reduce bias caused by uneven sequencing depth, the dataset was rarefied to 20,000 sequences per sample, a depth that exceeded the plateau of the rarefaction curves for all samples and maintained an average Good’s coverage of 99.09%, thereby capturing the majority of bacterial diversity while omitting minimal singletons. Taxonomic classification of representative OTU sequences was performed using the RDP Classifier (v2.2) against the 16 S rRNA gene database with a confidence threshold of 0.7 [27].

Based on the OTU data, rarefaction curves were generated, and alpha diversity indices (e.g., Simpson, Chao, Ace, and Shannon index) were calculated using Mothur (v1.30.1) [28]. Similarity among microbial communities in different samples was determined by principal coordinate analysis (PCoA) based on Bray-Curtis dissimilarity using the Vegan package (v2.5-3). Ordination was used to visualize similarities in microbial community structure among samples. Permutational multivariate analysis of variance (PERMANOVA), implemented in the same Vegan package, was applied to quantify the proportion of variation in community composition explained by soil type as well as to assess statistical significance. Prior to PERMANOVA, the assumption of homogeneity of multivariate dispersions was tested using the betadisper function in the Vegan package (permutations = 999). No significant differences in dispersion were detected among groups (*P* > 0.05), justifying the use of PERMANOVA to test for community composition differences. To visualize abundance dynamics of shared bacterial genera across plant growth stages, a co-occurrence network was constructed using Cytoscape [29]. The Kruskal–Wallis H test was used to identify taxa with significant differences in abundance among developmental stages. Redundancy analysis (RDA) was performed using the Vegan package (v2.4-3) in R (v3.3.1) to assess relationships between soil nutrient profiles and microbial community structure [30]. In addition, a heatmap based on Pearson correlation analysis was generated to visualize the strength and direction of associations between specific microbial taxa and soil nutrient factors. Functional prediction of bacterial communities was performed using FAPROTAX (Functional Annotation of Prokaryotic Taxa) v1.2.6. Based on the OTU representative sequences and their taxonomic assignments (confidence threshold ≥ 0.7), each OTU was mapped to known metabolic or biogeochemical functions using the default FAPROTAX database. The relative abundance of each predicted functional group was then calculated for each sample and compared across growth stages (seedling, flowering, maturity). All data analyses were conducted using the online tools available on the Majorbio Cloud Platform (https://www.majorbio.com/tools).

## Results

### Rhizosphere soil nutrient contents across different growth stages

**Fig. 1:**
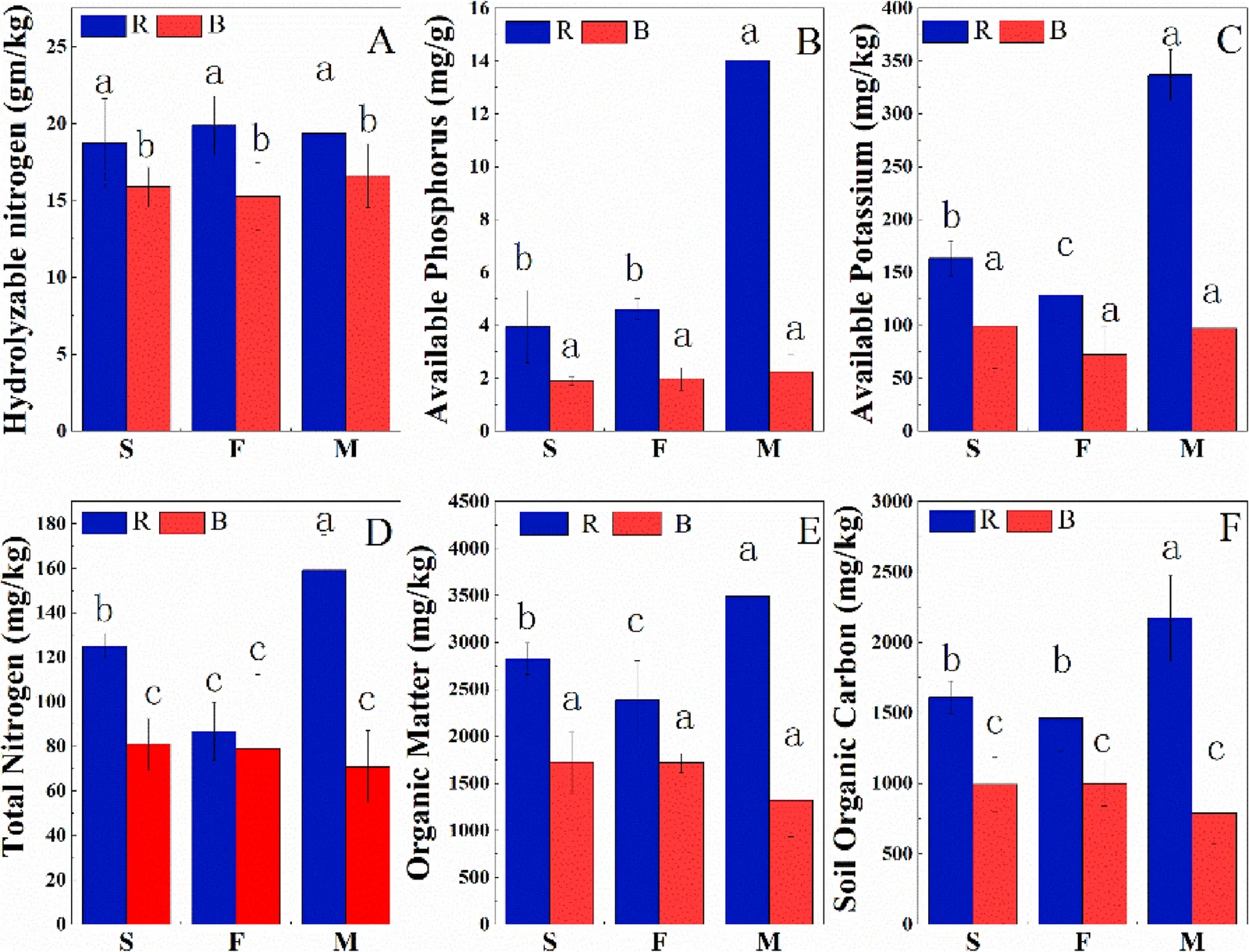
Rhizosphere nutrients of *L. racemosus* exhibit stage-specific dynamics. Hydrolyzable nitrogen (HN) remained stable throughout all growth stages, whereas available phosphorus (AP), total nitrogen (TN), available potassium (AK), organic matter (OM) and soil organic carbon (SOC) showed significant decreases at flowering and increases at maturity. (**A**) Hydrolyzable nitrogen, (**B**) available phosphorus, (**C**) available potassium, (**D**) total nitrogen, (**E**)organic matter, and (**F**) soil organic carbon. Different letters indicate statistically significant differences (Tukey’s HSD, *p* < 0.05; *n* = 10 per stage). S, seedling; F, flowering; M, maturity; R, rhizosphere; B, bulk soil

The rhizosphere soil of *L. racemosus* exhibited significantly higher concentrations of hydrolyzable nitrogen (HN: *p* = 0.023), AP (*p* = 0.008), organic matter (OM: *p* = 0.016), and Soil organic carbon (SOC, *p* = 0.011) than the bulk soil (Fig. 1). However, rhizosphere total nitrogen (TN), organic matter (OM), and Available potassium (AK) decreased significantly (TN: *p* = 0.036; OM: *p* = 0.022; AK: *p* = 0.032) during the flowering stage. In contrast, Available phosphorus (AP, *p* = 0.005), AK (*p* = 0.001), OM (*p* = 0.006), and Soil organic carbon (SOC, *p* = 0.026) increased significantly at the maturity stage (Fig. 1). Notably, HN remained unchanged throughout the entire *L. racemosus* growth period (Fig. 1A).

### Rhizosphere microbial community diversity across different growth stages

To address our first question regarding synchronous changes in community structure, we assessed bacterial diversity in the *L. racemosus* rhizosphere at the seedling, flowering, and maturity stages. After quality control and chimera removal, a total of 1,216,512 high-quality sequences were obtained from the assembled reads. These sequences were then clustered into OTUs at a 97% similarity threshold. Rarefaction based on the smallest sample size yielded 13,640 OTUs. Taxonomic classification identified 39 phyla, 126 classes, 297 orders, 507 families, and 1,006 genera.

**Fig. 2:**
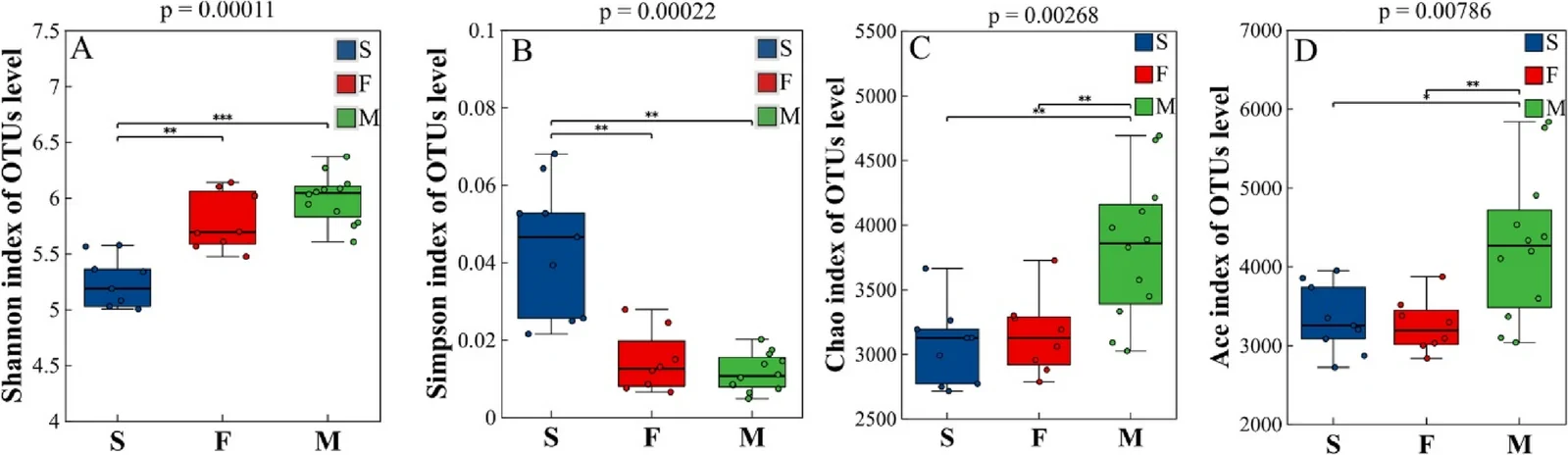
Alpha diversity of rhizosphere bacterial communities increases during *L. racemosus* development. Shannon and Simpson indices (**A**, **B**) were significantly higher at flowering and maturity than at seedling, while Chao and Ace indices (**C**, **D**) were highest at maturity, indicating increased diversity and richness. S, seedling; F, flowering; M, maturity. Kruskal Wallis H test with Dunn’s post hoc: *p < 0.05, **p < 0.01, ***p < 0.001. *n* = 10 per stage

Alpha diversity analysis showed that rhizosphere bacterial diversity varied among plant growth stages. The Shannon and Simpson indices indicated that bacterial diversity at the flowering and maturity stages was significantly higher than at the seedling stage (Kruskal-Wallis H test, *p* = 0.00469;Shannon: *p* = 0.00011; Simpson: *p* = 0.00022; Fig. 2A, B). In contrast, both the Chao and Ace indices indicated significantly higher richness only at the maturity stage (Kruskal-Wallis H test, *p* = 0.00469; Chao: *p* = 0.00268; Ace: *p* = 0.00268; Fig. 2C, D), indicating a significant accumulation of rhizosphere bacteria at the maturity stage.

**Fig. 3:**
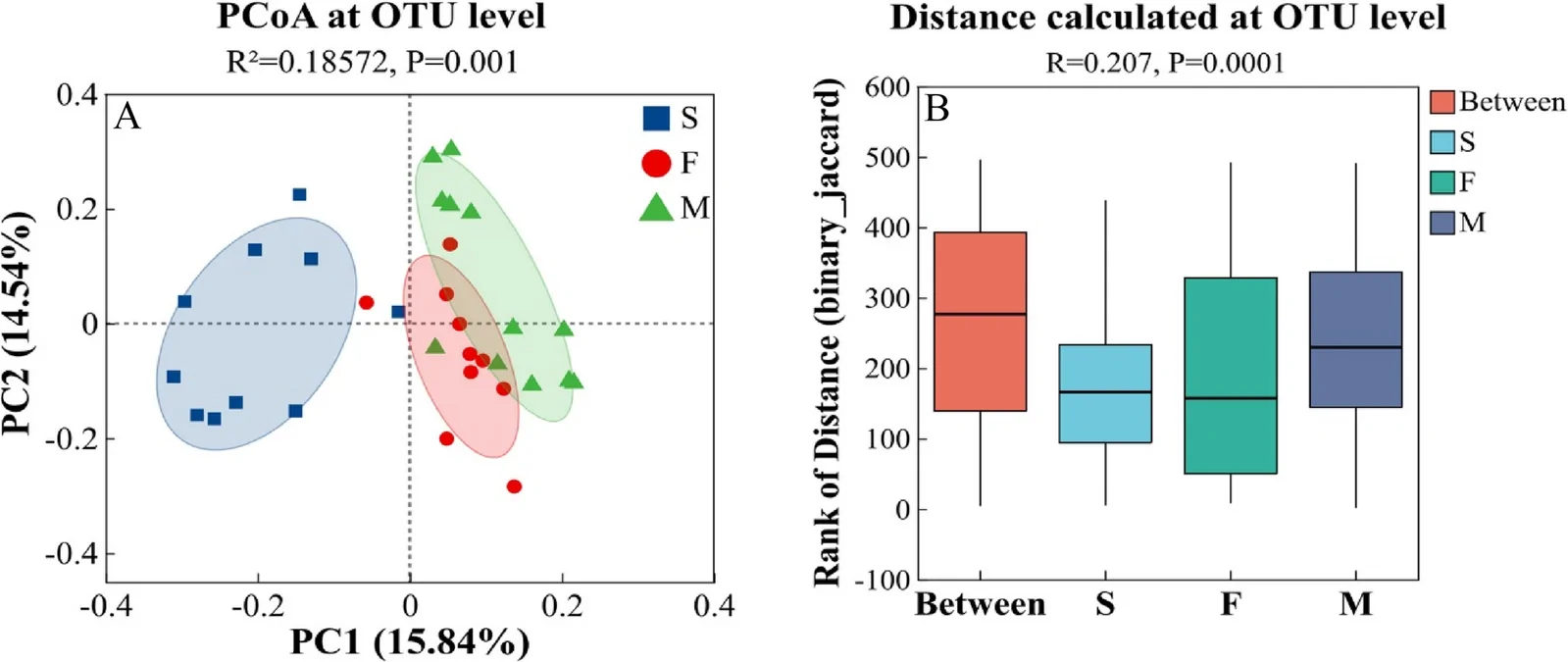
Beta diversity reveals distinct clustering of rhizosphere bacterial communities across growth stages. **A **Principal coordinate analysis (PCoA) based on Bray–Curtis dissimilarity shows clear separation of seedling (S), flowering (F), and maturity (M) samples. **B **Boxplot of inter group (Between) and intra group distances; higher Between distances indicate significant compositional differences among stages. ANOSIM, *p* = 0.001; PERMANOVA, R² = 0.207, *p* = 0.0001. *n* = 10 per stage

PCoA based on OTU-level data showed that rhizosphere bacterial communities from the seedling, flowering, and maturity stages formed distinct clusters, indicating high within-stage similarity. To further test for community differentiation, both ANOSIM and PERMANOVA were applied. ANOSIM confirmed a significant difference among stages (*p* = 0.001), and PERMANOVA likewise revealed a significant effect of growth stage on community composition (*p* = 0.0001), with the stage explaining 20.7% of the total variation (R² = 0.207; Fig. 3B). Together, these results demonstrate a clear shift in rhizobacterial structure during plant development, with growth stage playing a key role in shaping community assembly.

These results show that both bacterial diversity and richness increase with plant development, with the maturity stage harboring the highest bacterial richness, which aligns with the observed nutrient accumulation at that stage (see Fig. 1).

### Dynamics of key bacterial taxa across different growth stages

**Fig. 4:**
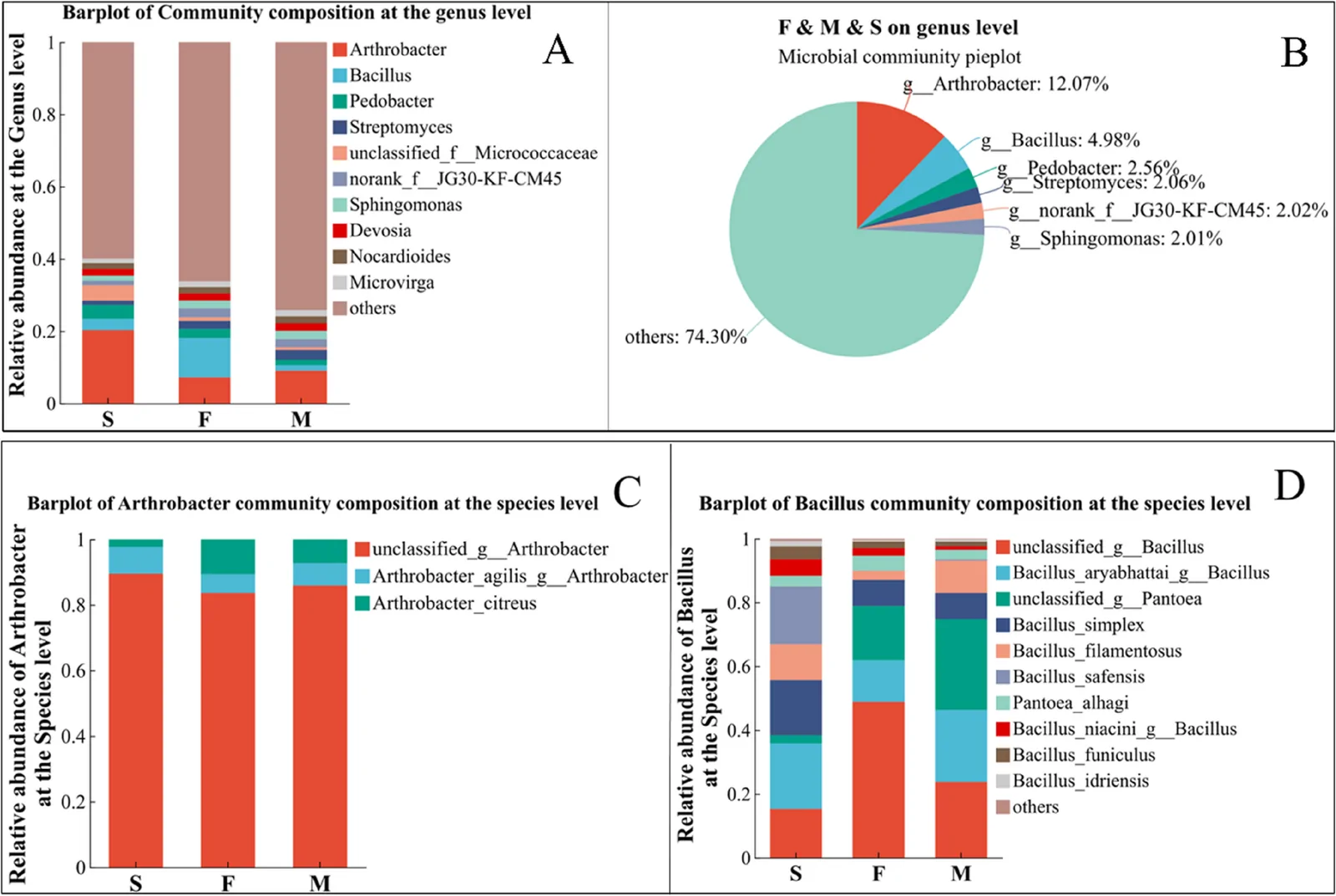
Stage-dependent shifts in dominant bacterial genera. *Arthrobacter* dominated all three stages (mean 12.7% relative abundance), whereas *Bacillus* increased sharply at flowering (from 3.1% to 10.9%) then declined at maturity. **A **Bar plot showing the top 10genera at the seedling (S), flowering (F), and maturity (M) stages. **B **Mean relative abundance of core bacterial genera across the growth cycle. **C **Species level composition of *Arthrobacter* across stages. **D **Species level composition of *Bacillus* across stages. Only genera with mean relative abundance >1% are shown; remaining taxa are grouped as “Others”. *n* = 10 per stage. Kruskal Wallis test, *p < 0.05, *p* < 0.01

Analysis of the rhizobacterial community composition of *L. racemosus* across different growth stages showed that *Arthrobacter* was the dominant genus at all three stages (Fig. 4A, B). Specifically, *Arthrobacter* had a mean relative abundance of 12.7%, ranging from 20.3% at the seedling stage to 7.2% at flowering, and then increased slightly to 9.7% at maturity. Despite the pronounced decline during flowering, *Arthrobacter* remained at notably high relative abundance. The decrease in *Arthrobacter* abundance at the flowering stage coincided with a substantial increase in *Bacillus*, which rose from 3.1% at the seedling stage to 10.9% at flowering, establishing it as a major dominant genus during this period; its abundance then dropped to 1.5% at the maturity stage (Fig. 4A, B).

Species-level analysis of *Arthrobacter* and *Bacillus* revealed distinct temporal dynamics across plant developmental stages. Within the genus *Arthrobacter*, an unclassified species (*unclassified_g__Arthrobacter*) dominated the community at the seedling, flowering, and maturity stages, accounting for more than 80% of the relative abundance (Fig. 4C). *Arthrobacter agilis* and *Arthrobacter citreus* were also present at relatively high proportions (Fig. 4C). In contrast, the increase in *Bacillus* abundance during the flowering stage was primarily driven by a marked increase in an unclassified *Bacillus* species (*unclassified_g__Bacillus*). Meanwhile, *Bacillus aryabhattai*, *Bacillus filamentosus*, and *Bacillus simplex* maintained relatively stable abundances across all stages (Fig. 4D).

Co-occurrence network analysis further revealed growth stage-dependent variations in the abundance of bacterial genera (Fig. S1). Consistent with earlier results, *Arthrobacter* maintained high relative abundance throughout the seedling, flowering, and maturity stages, confirming its role as a stable dominant genus in the *L. racemosus* rhizosphere. *Bacillus* reached its peak abundance during the flowering stage. At the same time, the abundances of *Pantoea* and *Massilia* also increased significantly, accounting for 3.0% and 1.9% of the community, respectively.

Analysis of differentially abundant taxa further showed that *Arthrobacter* consistently exhibited high abundance across all developmental stages, with its relative abundance at the seedling stage being significantly higher than at the flowering or maturity stages. In addition, *Pedobacter*, *TM7a*, and *Planomicrobium* were significantly more abundant at the seedling stage than at the other two stages. During the flowering stage, in addition to *Bacillus*, the abundances of *Pantoea*, *Massilia*, and *Sphingomonas* were significantly elevated, establishing them as dominant taxa for this period. In contrast, the maturity stage was characterized by sustained high relative abundances of *Arthrobacter*, *norank_f__JG30-KF-CM45*, *Sphingomonas*, *Devosia*, and the *Allorhizobium-Neorhizobium-Pararhizobium-Rhizobium* group (Fig. S2).

Consistent with our first hypothesis, *Arthrobacter* maintained dominance across all stages, but its relative abundance declined at flowering, while *Bacillus* showed a sharp, transient increase—a pattern that directly reflects a shift from a core stability‑providing taxon to a functional, demand‑responsive taxon.

### Functional characterization of key bacterial communities at different growth stages

**Fig. 5:**
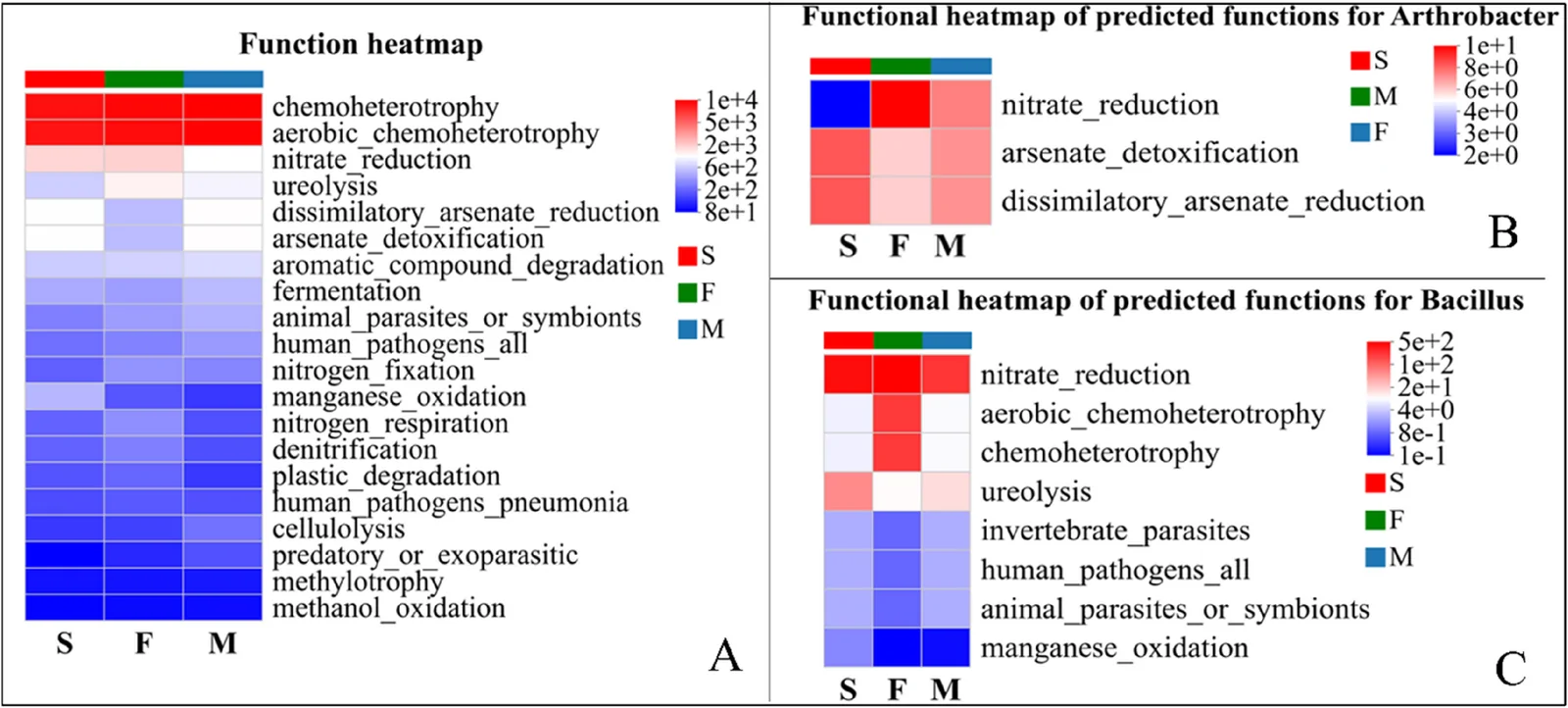
FAPROTAX based functional predictions of the rhizosphere microbiota. **A **Predominant functions across seedling (S), flowering (F), and maturity (M) stages: aerobic chemoheterotrophy, chemoheterotrophy, and nitrate reduction are core. Ureolysis was significantly enriched at flowering. **B **Functional prediction for the genus *Arthrobacter* highlights nitrate reduction as its core function. **C **Functional prediction for *Bacillus* shows enhanced aerobic chemoheterotrophy and chemoheterotrophy at flowering. Color gradient indicates variation in abundance across samples. *n* = 10 per stage

FAPROTAX-based functional profiling identified aerobic chemoheterotrophy, chemoheterotrophy, and nitrate reduction as the core functions of the bacterial community (Fig. 5A). In addition, ureolysis was significantly more abundant during the flowering stage than during the seedling and maturity stages.

As mentioned, the genus *Arthrobacter*

dominated the rhizosphere community across growth stages. Notably, the core function of the *Arthrobacter* genus was nitrate reduction, followed by arsenate detoxification and reduction (Fig. 5B). In contrast, *Bacillus* abundance increased significantly at the flowering stage. Functional prediction for the *Bacillus* genus revealed not only a strong potential for nitrate reduction but also a significant enhancement of aerobic chemoheterotrophy and chemoheterotrophy at the flowering stage (Fig. 5C).

### Associations between microbial communities and soil factors

**Fig. 6:**
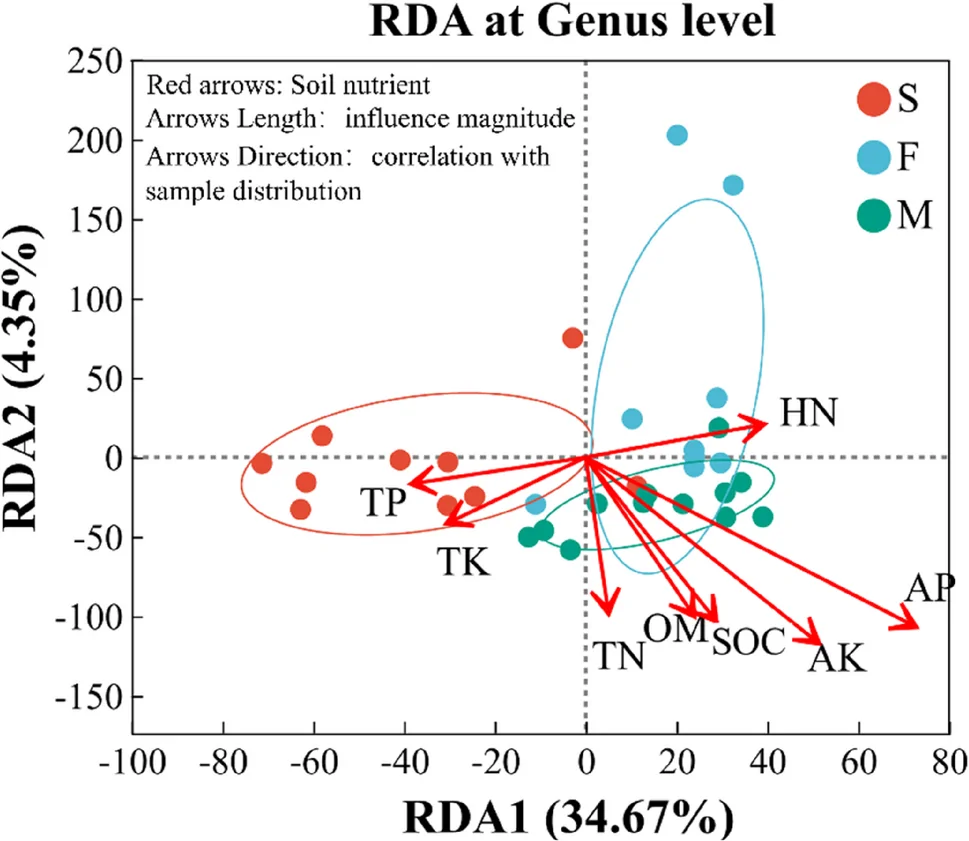
Redundancy analysis (RDA) relating rhizosphere bacterial community structure to soil nutrient variables. Maturity (M) samples cluster closely with vectors for AP, AK, TN, and soil organic carbon (SOC), indicating strong positive associations. Red arrows: length indicates influence magnitude; direction indicates correlation with sample distribution. SOC, soil organic carbon; OM, organic matter; HN, hydrolyzable nitrogen; TN, total nitrogen; AN, available nitrogen; TP, total phosphorus; AP, available phosphorus; TK, total potassium; AK, available potassium. *n* = 10 per stage

To test our second hypothesis—that co‑occurrence patterns among bacterial genera are associated with nutrient dynamics— we performed RDA and Spearman correlation analysis. The RDA results (Fig. 6) showed that samples from the maturity stage were closely aligned with the vectors for AP, AK, TN, and soil organic carbon (SOC). This alignment indicates a strong association between the rhizobacterial community structure at the maturity stage and the enrichment of these soil nutrients.

**Fig. 7:**
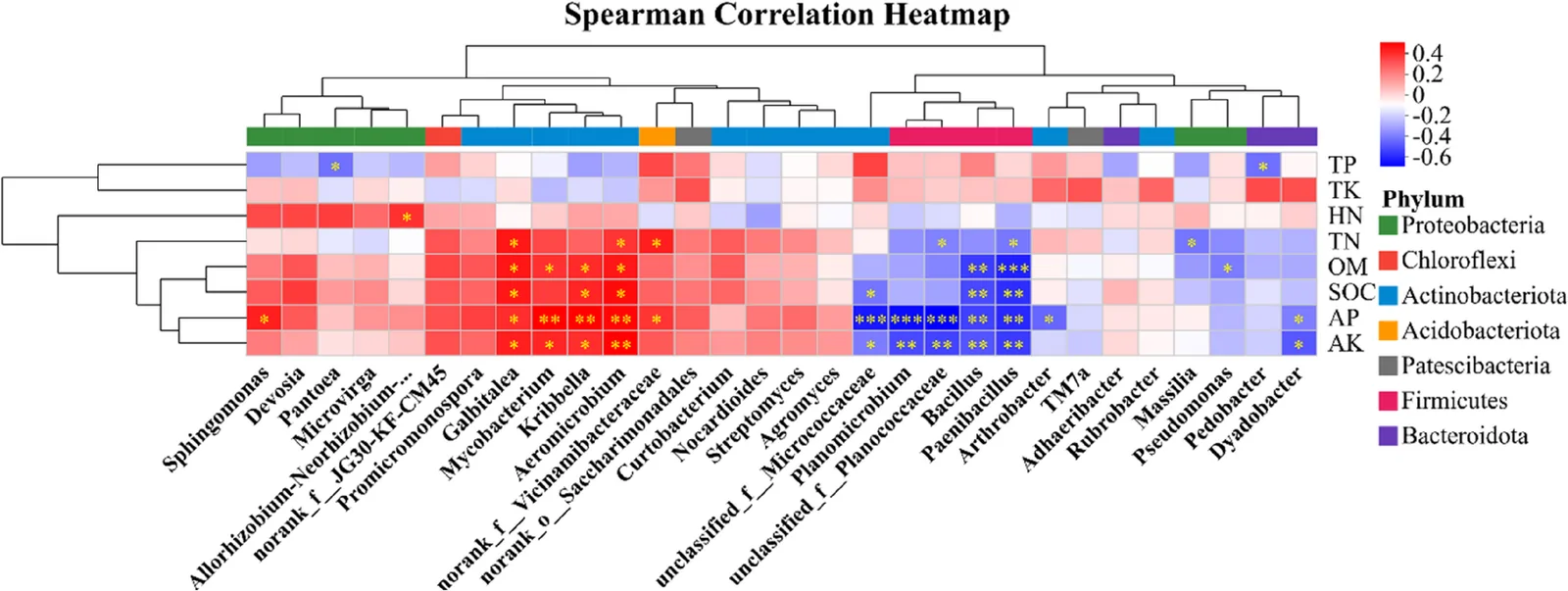
Heatmap of Spearman correlations between the top 30 bacterial genera and soil nutrients. *Bacillus* abundance is significantly negatively correlated with AP, AK, and SOC. At maturity, *Sphingomonas*, *Mycobacterium*, *Kribbella*, and *Aeromicrobium* show significant positive correlations with AP and AK; *Kribbella* and *Aeromicrobium* are also positively correlated with SOC. Color intensity: Pearson correlation coefficient (R). *0.01 < *p* ≤ 0.05; **0.001 < *p* ≤ 0.01; ***p ≤ 0.001. *n* = 10 per stage

To further elucidate the roles of specific bacterial genera in nutrient dynamics, Spearman correlation analysis was conducted on the top 30 most abundant genera (Fig. 7). As shown in Fig. 7, the abundance of *Bacillus* was significantly negatively correlated with AP, AK, and SOC, supporting the idea that its flowering‑stage enrichment is linked to nutrient depletion. In contrast, at maturity, *Sphingomonas*, *Mycobacterium*, *Kribbella*, and *Aeromicrobium* showed significant positive correlations with AP, AK, and SOC, illustrating how distinct microbial alliances are associated with different nutrient phases.

## Discussion

A growing body of evidence has established that plant developmental stage is a key driver of rhizosphere microbial community succession [7, 8]. For example, in wheat, Chen et al. (2019) documented distinct shifts in bacterial community structure across seedling, heading, and grain-filling stages [7]. In maize and tobacco, similar stage-dependent changes in microbial diversity and dominance have been reported [12, 15]. These studies collectively indicate that plants actively recruit specific microbial taxa at different phenological phases to meet changing nutritional demands. Consistent with this view, our results from *L. racemosus* reveal a clear successional pattern from a *Arthrobacter*-dominated seedling stage to a *Bacillus*-enriched flowering stage, followed by a re-established *Arthrobacter*-associated community at maturity. Below, we discuss the functional implications of this successional dynamics in the context of nutrient cycling.

### *Arthrobacter* as a core beneficial taxon and stabilizer of the *L. racemosus* rhizosphere

Our results show that *Arthrobacter* dominated the rhizosphere across all three stages, with a stable relative abundance (mean 12.7%) and a predicted core function of nitrate reduction. This persistent dominance, coupled with the unchanged hydrolyzable nitrogen content throughout the plant’s life cycle, is consistent with the hypothesis that *Arthrobacter* acts as a nitrogen‑buffering system.

Actinobacteriota are widely distributed in soil environments characterized by low water content, high organic matter, and slightly alkaline conditions [31, 32], and they play key roles in nitrogen cycling. In this study, Actinobacteriota were significantly enriched in the *L. racemosus* rhizosphere, indicating that this group may contribute to plant growth and environmental adaptation. Within this phylum, *Arthrobacter* has been reported as a dominant rhizosphere taxon of desert plants [33]. Members of this genus exhibit broad carbon source utilization and strong environmental adaptability. Many strains are recognized as plant growth-promoting rhizobacteria (PGPR), with functions that include phosphorus solubilization, nitrogen fixation, potassium release, ACC deaminase production, antibiotic synthesis, and phytohormone secretion [34–36]. Of particular importance is their extensive nitrogen metabolic capacity. Certain strains can decompose and mineralize organic nitrogen (e.g., proteins and amino acids) through extracellular enzyme secretion, directly supplying plants with absorbable ammonium (NH₄⁺) [37–39]. In addition, some strains participate in nitrification and denitrification and may even possess nitrogen-fixing potential, thereby regulating nitrogen forms through multiple pathways to support plant nitrogen nutrition [37, 40, 41].

Consistent with our first hypothesis, *Arthrobacter* maintained dominance in the *L. racemosus* rhizosphere across all growth stages, but its relative abundance and potential functional roles shifted in ways that corresponded to stage‑specific changes in available nitrogen pools. At the seedling stage, the absolute dominance of *Arthrobacter*, combined with its strong aerobic chemoheterotropic, chemoheterotrophic, and nitrate reduction capacities, likely constituted a core mechanism supporting a steady nitrogen supply and the establishment of a basic nutrient cycle. This pattern aligns with the persistently high and stable level of HN (available nitrogen) observed in the rhizosphere throughout the early growth period. At the flowering stage, although the relative abundance of *Arthrobacter* declined, its versatile metabolism of both organic and inorganic nitrogen (e.g., nitrate), together with its potential nitrogen‑fixing capacity, likely enabled it to maintain functional importance under shifting nitrogen forms. *Arthrobacter* likely maintained functional importance through its versatile metabolism of both organic and inorganic nitrogen (e.g., nitrate), together with its potential nitrogen-fixing capacity, enabling persistence under shifting nitrogen forms. By the maturity stage, *Arthrobacter*, persisted as a keystone taxon and likely acted synergistically with other nutrient-mobilizing genera such as *Sphingomonas* and *Devosia* [42, 43], continuing to promote nitrogen release via chemoheterotrophy and nitrate reduction. As plant nitrogen demand declined at this stage, sustained microbial activity probably contributed to the observed rhizosphere nutrient enrichment.

Taken together, these findings support our first hypothesis: the relative abundance and functional expression of a core taxon (*Arthrobacter*) varied across growth stages, and these variations were closely linked to stage‑specific changes in soil nitrogen pools. Based on these findings, we propose that a core bacterial microbiome centered on *Arthrobacter* forms a robust microbial buffering system. This system may mitigate nitrogen fluctuations driven by plant uptake and extreme environmental variability in desert soils through rapid transformation of organic and inorganic nitrogen sources, thereby maintaining dynamic stability of available nitrogen in the *L. racemosus* rhizosphere. Such buffering capacity—likely derived from its abilities to mineralize organic nitrogen, reduce nitrate, and potentially fix atmospheric nitrogen—may represent a key ecological strategy supporting a stable nitrogen supply in harsh desert environments. However, direct in situ evidence for this buffering mechanism is still needed.

### Flowering‑stage enrichment of *Bacillus* represents a “rapid‑investment” strategy to meet peak nutrient demand

The significant increase in rhizosphere *Bacillus* abundance during the flowering stage may be potentially correlated with shifts in soil nutrients. *Bacillus*, a genus within the phylum *Firmicutes*, exhibits strong environmental adaptability, allowing it to survive under stress conditions such as nutrient limitation, drought, and high temperatures. It plays an important role in global biogeochemical cycles, including carbon and nitrogen cycling [44]. As typical PGPR, many *Bacillus* strains can synthesize and secrete phytohormones such as auxins and cytokinins, which stimulate cell division and elongation and thereby enhance root and shoot growth [45]. In addition, *Bacillus* species contribute to plant nutrition by fixing atmospheric nitrogen, solubilizing inorganic phosphorus, and mobilizing potassium, converting immobilized soil nutrients into plant-available forms [46, 47]. Specifically, *Bacillus* species solubilize inorganic phosphorus through the secretion of organic acids and acid phosphatases and mineralize organic phosphorus via phytase secretion, leading to rapid increases (within days to weeks) in available phosphorus (H₂PO₄⁻/HPO₄²⁻) [48, 49]. Concurrently, metabolites such as organic acids and amino acids released by *Bacillus* can weather potassium-bearing minerals and release plant-available potassium ions (K⁺) [50, 51]. In the nitrogen cycle, *Bacillus* is a key driver in the transformation of organic nitrogen to inorganic forms, as secreted proteases efficiently degrade organic nitrogen and rapidly generate ammonium (NH₄⁺), thereby increasing soil available nitrogen [47, 52].

Consistent with our first hypothesis, we observed a significant increase in the relative abundance of *Bacillus* in the *L. racemosus* rhizosphere during the flowering stage, followed by a marked decline at maturity. This dynamic pattern is likely linked to stage‑specific nutrient demands: flowering represents a critical phase with peak requirements for nitrogen, phosphorus, and potassium, whereas maturity involves reduced nutrient uptake. The enrichment of *Bacillus* at flowering therefore likely reflects active recruitment by the plant, serving as a microbial “rapid‑response force” to mobilize soil nutrients. It is well established that cultured *Bacillus* strains can solubilize phosphorus, mobilize potassium, and produce phytohormones. In our study, FAPROTAX‑based predictions further suggest that *Bacillus* in the *L. racemosus* rhizosphere may enhance aerobic chemoheterotrophy and nitrate reduction during flowering. Collectively, these functions could support plant nutrition by rapidly increasing available phosphorus and potassium, as well as converting organic nitrogen to ammonium, thereby meeting the high demands of flowering. Although causal evidence remains to be established, the tight correspondence between the stage‑specific abundance shift of *Bacillus* and concurrent changes in soil nutrient pools supports our first hypothesis and provides a testable model for future mechanistic studies—for example, via metagenomics or strain isolation to validate the predicted functions.

Despite the enrichment of *Bacillus*, no significant changes in available nitrogen or available phosphorus were detected in the *L. racemosus* rhizosphere during flowering. In contrast, both available potassium and total nitrogen decreased significantly, resulting in an overall decline in total rhizosphere nutrient levels. We hypothesize that this pattern reflects the high nutrient demand during flowering, whereby nutrients mobilized by microorganisms are rapidly taken up by the plant, creating a nutrient-limited rhizosphere and preventing nutrient accumulation. Thus, the enrichment of *Bacillus* during flowering likely represents a demand-driven recruitment strategy by *L. racemosus*, functioning as a temporary and highly efficient microbial support system.

Notably the flowering-stage rhizosphere of *L. racemosus* was enriched not only in *Bacillus*, but also in genera such as *Pantoea*, *Massilia*, and *Sphingomonas*. Previous studies have shown that many *Pantoea* strains possess phosphorus-solubilizing capacity, whereas *Sphingomonas* can enhance nitrogen and phosphorus cycling in the rhizosphere [53, 54]. These findings suggest that a multispecies microbial consortium may act synergistically to support the high-intensity nutrient demands of *L. racemosus* during flowering.

This pattern is consistent with our first hypothesis: a functional taxon is transiently enriched to meet stage‑specific high demand for phosphorus and potassium. However, the lack of net accumulation of available nutrients at flowering suggests that the mobilized nutrients are rapidly taken up by the plant, reflecting an “investment” rather than “storage” strategy.

### Maturity‑stage shift from investment to “harvest”: synergistic microbial networks drive delayed nutrient accumulation

Co-occurrence patterns among bacterial genera might be associated with the observed nutrient dynamics. Upon entering the maturation stage, although the relative abundance of *Bacillus* decreased sharply and that of *Arthrobacter* showed only a slight recovery, the contents of available phosphorus (AP), available potassium (AK), and organic carbon (SOC) increased significantly. This seemingly counterintuitive pattern is explained by our second hypothesis: co-occurrence patterns among bacterial genera drive sustained nutrient mobilization.

First, the extensive proliferation and metabolic activity of *Bacillus* during the flowering stage may have exerted a lasting influence on the rhizosphere microenvironment, creating favorable conditions for the colonization and functional expression of other microorganisms. This mechanism reflects a time-lag effect of microbial function on nutrient availability.

Second, co-occurrence patterns among bacterial genera appear to drive sustained nutrient mobilization during maturation. Our results showed that taxa such as norank_f__JG30-KF-CM45, *Sphingomonas*, *Devosia*, and the *Allorhizobium-Neorhizobium-Pararhizobium-Rhizobium* group maintained relatively high abundances during this stage. Correlation analysis further revealed that *Devosia* was significantly positively correlated with available phosphorus and organic matter, while *Sphingomonas* was also significantly positively correlated with available phosphorus. These positive correlations illustrate how co-occurring taxa may form a functionally complementary microbial network, collectively promoting sustained nutrient activation and accumulation even after the decline of early-stage dominants like *Bacillus*. This synergy represents a delayed nutrient enrichment effect arising from interspecific co-occurrence patterns.

Additionally, as the plant transitions into the maturation stage, its physiological and metabolic activities slow, leading to reduced nutrient uptake demand and rate. Consequently, nutrients mobilized by the microbial community are no longer rapidly absorbed by the plant and are more likely to accumulate within the rhizosphere, further contributing to the observed enrichment.

Overall, the co‑occurrence of genera such as *Devosia* and *Sphingomonas*—positively correlated with available nutrients—is linked to nutrient dynamics at the maturation stage. Specifically, microbial synergy within a complementary network can drive delayed nutrient enrichment, even as previously dominant taxa decline. Thus, the positive correlations between these taxa and available nutrients at maturity provide correlational evidence supporting hypothesis ii.

### Nutrient-microbe interplay in the *L. racemosus* rhizosphere: Integrated model and future directions

**Fig. 8:**
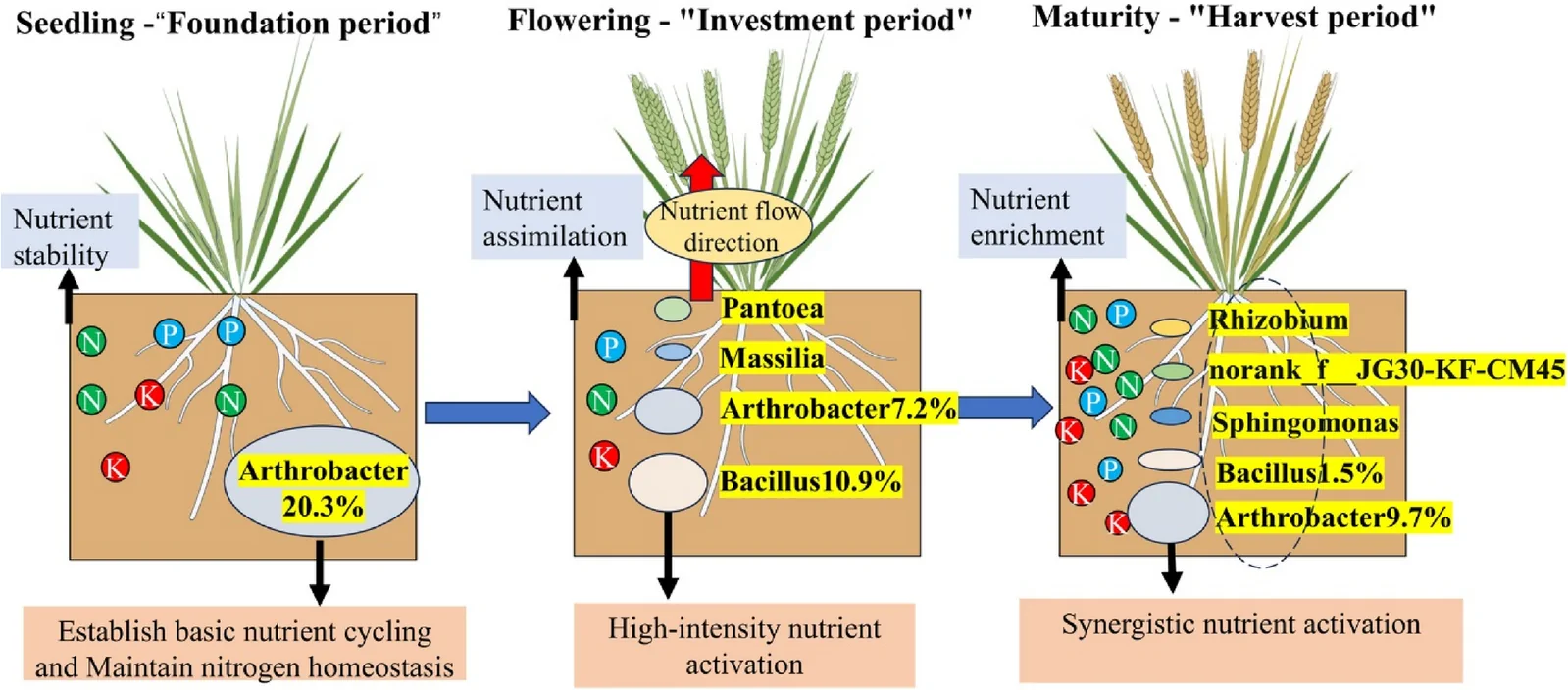
Conceptual model of rhizosphere microbial and nutrient dynamics across L. racemosus developmental stages. Seedling: “Foundation phase” – Arthrobacter centered system maintains nitrogen stability. Flowering: “Investment phase” – recruitment of Bacillus mobilizes nutrients for reproduction, depleting the soil nutrient pool. Maturity: “Harvest phase” – reduced plant demand and synergistic microbial actions lead to nutrient enrichment

Based on the results described above, we developed a conceptual model (Fig. 8) that integrates and tests our two exploratory hypotheses using observational data. First, the relative abundances of core and functional taxa vary across growth stages and are linked to stage‑specific changes in soil nutrient pools—we observed distinct successional patterns: *Arthrobacter* dominated the seedling stage, *Bacillus* was enriched at flowering, and a synergistic network of taxa (e.g., *Devosia*, *Sphingomonas*) persisted into maturation. These shifts corresponded directly to changes in nitrogen stability (seedling), rapid nutrient mobilization and depletion (flowering), and delayed nutrient accumulation (maturation). Second, the co‑occurrence patterns among bacterial genera are associated with nutrient dynamics—correlation analyses revealed positive associations between *Devosia*,* Sphingomonas*, and available phosphorus/organic matter during the maturation stage, illustrating how microbial synergy can lead to delayed nutrient enrichment even after the decline of early‑stage dominants.

Together, these findings support a three‑phase conceptual model of the *L. racemosus* rhizosphere. The seedling stage represents a “foundation phase”, during which a rhizosphere system centered on *Arthrobacter* may contribute to maintaining nitrogen stability, as suggested by its consistent dominance and predicted nitrate reduction capacity (direct evidence still required). As the plant enters the flowering stage, nutrient demand increases sharply, initiating an “investment phase”. This stage is characterized by the pronounced recruitment of *Bacillus*, which actively mobilizes soil nutrients to support reproductive growth, leading to rapid depletion of the soil nutrient pool and a marked decline in rhizosphere nutrient content. The maturation stage can be viewed as a “harvest phase”, during which plant nutrient demand declines and microbial communities act synergistically to enhance nutrient accumulation beyond plant uptake, resulting in pronounced nutrient enrichment in the rhizosphere.

While previous frameworks have recognized plant developmental stage as a driver of rhizosphere microbial succession (e.g., Chen et al., 2019), our conceptual model extends these by explicitly differentiating between a persistent core taxon (*Arthrobacter*) and a transient functional genus *(Bacillus*). Furthermore, it introduces a triphasic sequence—foundation, investment, harvest—that links microbial recruitment strategies to stage‑specific nutrient demand and accumulation in a desert perennial. This integrative view has not, to our knowledge, been previously proposed. This model answers our overarching question: *L. racemosus* recruits distinct microbial alliances at different phenological phases—a persistent *Arthrobacter*-based system for nitrogen buffering (seedling), a transient *Bacillus*-enriched community for rapid nutrient mobilization (flowering), and a synergistic network for delayed nutrient accumulation (maturity).

### Limitations and future directions

We emphasize that the proposed “foundation-investment-harvest” model is a hypothesis-driven framework synthesized from correlational data, not a demonstration of causation. In this study, we acknowledge several specific limitations. First, our findings are descriptive and correlational; bacterial functions were inferred solely from the FAPROTAX database, providing only predicted metabolic potentials rather than direct evidence of causality. Second, while we interpret the stage-specific enrichment of *Bacillus* as a potential plant-mediated recruitment strategy, we lack direct measurements of root exudates or other plant signals; alternative explanations (e.g., passive microbial growth due to altered soil conditions) cannot be ruled out. Third, and most importantly, the study was conducted at a single desert site (Kalamaili Nature Reserve) within one growing season, which limits the generalizability of our conclusions to other populations or years.

Given these limitations, the primary value of our model lies in generating testable predictions for future mechanistic studies. For example, direct causal relationships—such as whether *Arthrobacter* drives nitrogen stability, whether *Bacillus* causally mediates nutrient depletion at flowering, and whether synergistic co-occurrence networks are necessary for the delayed enrichment observed at maturation—should be rigorously tested using gnotobiotic systems, knockout mutants, or isotopic tracing. More broadly, future work should combine multi-site, multi-year sampling with metagenomics, pure culture isolation, genome sequencing, plant inoculation assays under controlled conditions, and root exudate metabolomics. Such approaches will help establish causal relationships and assess the environmental robustness of the observed stage-dependent rhizosphere succession patterns. Our findings are therefore hypothesis-generating, providing correlational evidence that warrants rigorous experimental testing.

## Conclusion

This study set out to answer how rhizosphere microbial succession is linked to nutrient dynamics across the life cycle of a desert plant. We conclude that *Leymus racemosus* employs a stage‑dependent recruitment strategy: *Arthrobacter* provides a stable nitrogen‑buffering system throughout all stages; *Bacillus* is transiently enriched at flowering to mobilize nutrients for reproductive growth; and at maturity, synergistic networks (e.g., *Sphingomonas*, *Devosia*) drive delayed nutrient accumulation. These findings are consistent with our two hypotheses and provide a testable framework (foundation‑investment‑harvest model) for future mechanistic studies. However, our interpretations are primarily derived from correlation and functional prediction analyses, and the current dataset is limited to a single site and a single growing season. Future research should integrate multi‑site, multi‑year sampling with strain isolation, inoculation assays, root exudate metabolomics, and metagenomics to establish causality.

## Supplementary Information

Supplementary Material 1.

Supplementary Material 2.

Supplementary Material 3.

## Data Availability

Datasets generated during the current study are available in the NCBI repository under Bioproject number PRJNA1241221 ( https://dataview.ncbi.nlm.nih.gov/object/PRJNA1241221 ; accessed 1 November 2025). The corresponding accession numbers for this submission are SRR35918480-SRR35918497.

## Abbreviations

S: Seedling
F: Flowering
M: Maturity
TN: Total nitrogen
AN: Available nitrogen
AP: Available phosphorus
TK: Total potassium
AK: Available potassium
SOC: Soil organic carbon
OTUs: Operational taxonomic units
RDA: Redundancy analysis
R: Rhizosphere Soil
B: Bulk soil
PCoA: Principal Co-ordinates Analysis
PERMANOVA: Permutational Multivariate Analysis Of Variance
FAPROTAX: Functional Annotation of Prokaryotic Taxa
PGPR: Plant growth-promoting rhizobacteria
ACC: Acetyl - CoA carboxylase
