# Supplementary material for: Stage-specific rhizosphere microbial succession is associated with nutrient cycling in the desert plant Leymus racemosus (Lam.) tzvelev
Source: BMC Plant Biol. 2026 Jun 20;26:1501. doi: 10.1186/s12870-026-09294-z (PMC13540912; doi:10.1186/s12870-026-09294-z)
Supplement: Supplementary file 1 [file 12870_2026_9294_MOESM1_ESM.pdf]

附表 2:

自治区重点保护野生植物采集申请表

申请单位、个人	新疆农业大学生命科学学院 孙玉芳			
单位地址、邮编	乌鲁木齐市沙依巴克区农大东路 311 号			
联系人	孙玉芳	联系电话	15999168641	
采 集 目 的	科学研究			
采 集 地 点	新疆省（自治区、直辖市） 阿勒泰 地区（市） 卡拉麦里有蹄类野生动物自然保护区<br>卡拉麦里有蹄类野生动物自然保护区，中心坐标位于：45° 14′ 47″ N，89° 4′ 56″ E，海拔：953.54m 的区域			
物种中文名	学 名	保护级别	采集部位	采集数量
大赖草	Leymus racemosus (Lam.) Tzvelev	自治区二级	根际土壤和部分植株以及种子	根际土壤：300g，植株：50 株，种子：500g
采集时间： 自 2026 年 5 月 1 日至 2028 年 8 月 30 日				

申请单位、个人签字（签章）

孙玉芳

2026 年 3 月 3 日

县级、地州市级行政主管部门意见：

经办人：[illegible]

审核人：[illegible]

签发人：[illegible]

2026 年 3 月 5 日

（加盖公章）

说明：1、采集地点需注明至村或林班。2、采集数量填写株数、枝数或重量（kg）。
