# Supplementary material for: Stage-specific rhizosphere microbial succession is associated with nutrient cycling in the desert plant Leymus racemosus (Lam.) tzvelev
Source: BMC Plant Biol. 2026 Jun 20;26:1501. doi: 10.1186/s12870-026-09294-z (PMC13540912; doi:10.1186/s12870-026-09294-z)
Supplement: Supplementary file 2 [file 12870_2026_9294_MOESM3_ESM.zip › supplementary file/figure caption.docx]

**FigureS1(5). Co‑occurrence network illustrating stage‑specific relative abundances of bacterial genera.** Each node represents a genus; colored sectors indicate relative abundance at seedling (purple), flowering (green), and maturity (yellow). *Arthrobacter* remains abundant across all stages, while *Bacillus* peaks at flowering. n = 10 per stage.

**FigureS2(6).** **Differentially abundant rhizosphere bacterial genera across growth stages.** Arthrobacter showed high abundance at all stages (significantly highest at seedling). *Pedobacter*, TM7a, and *Planomicrobium* were enriched at seedling; *Bacillus*, *Pantoea*, *Massilia*, and *Sphingomonas* at flowering; and norank_f_JG30‑KF‑CM45, *Devosia*, and *Allorhizobium‑Neorhizobium‑Pararhizobium‑Rhizobium* group at maturity. Kruskal‑Wallis H test with FDR correction, p < 0.05. n = 10 per stage.
